# Inclusion in Motion: Promoting Equitable Physical Activity and Health in Childhood and Adolescence

**DOI:** 10.3390/children12070942

**Published:** 2025-07-17

**Authors:** Vidar Sandsaunet Ulset, Luca Oppici, Karin Hamre, James Robert Rudd, Annett Victoria Stornæs, Heidi Marian Haraldsen, Reidar Säfvenbom

**Affiliations:** 1 Development in Context Research Group, Department of Teacher Education and Outdoor Studies, Norwegian School of Sport Sciences, Sognsveien 220, 0863 Oslo, Norway; lucao@nih.no (L.O.);; 2 Monash University European Research Foundation ETS, 59100 Prato, Italy; 3 Department of Sports, Diversity and Public Health, Akershus County Council, Schweigaards gate 4, 0185 Oslo, Norway; karinhamr@afk.no

**Keywords:** inclusion, physical activity, marginalization, health disparities, developmental systems theory, resilience, interdisciplinary research, life course, equity in education, movement contexts

## Abstract

Inclusion in play, physical education, outdoor life, organized sports, and other movement-based activities can promote resilience and support physical, emotional, and social well-being. These arenas are particularly important for reducing health disparities and preventing social marginalization across the lifespan. Yet, children and adolescents from vulnerable or disadvantaged backgrounds encounter persistent barriers to participation, rooted in broader inequalities related to their socioeconomic position, disability, gender, ethnicity, and access to supportive environments. This perspective outlines how inclusive movement contexts, when informed by developmental systems theory and resilience frameworks, can interrupt trajectories of marginalization and promote long-term equity in health, education, and work inclusion. We emphasize the need for interdisciplinary approaches, combining longitudinal and qualitative methods, to uncover how vulnerability and participation interact dynamically over time. By integrating insights from developmental science, education, public health, and spatial ecology, we identify strategic pathways for research and action. Addressing these challenges requires coordinated efforts across sectors and stakeholders to co-create inclusive, context-sensitive interventions.

## 1. Introduction

The importance of reducing social disparities in health and well-being and preventing marginalization is a key endeavor within the United Nation’s sustainable development goals [1]. While there has been a steady decline in physical activity (PA) and participation in various movement contexts, in the past 50 years, in the overall population [2], the decline may be more emphasized in certain populations. Girls, women, elderly people, people of a low socioeconomic position, people with disabilities and chronic diseases, marginalized populations, and the inhabitants of rural communities engage less in PA and have less access to safe, suitable, and affordable spaces and places in which to be physically active [3,4,5,6,7]. There is a need for interventions that target the roots of the problem.

Interventions can be implemented in different settings, including nature, physical education (PE), and formal and organized sports as well as informal and negotiable movement contexts. The importance of the access to nature for PA has been stressed by the World Health Organization (WHO), which has defined minimum standards for neighborhood greenspace [8]. Yet, according to WHO standards, more than one third of all citizens in European cities do not have access to adequate greenspaces [9]. A recent report from our department highlights the importance of equal access to nature-based recreation as potential means of reducing social disparities and preventing marginalization [5]. Unfortunately, similar disparities are evident in organized sports and education. Social class and limited economical resources as well as more relational challenges are considered major barriers for participation in organized sports [10,11,12]. Globally, PE often prioritizes competitive sports, which may disadvantage students without prior experience, particularly those with disabilities, limited parental support, or marginalized socioeconomic or cultural backgrounds [13,14,15,16,17], and the need for inclusive PE for children with various challenges to participate is evident [18]. PE curriculums often advocate for adaptability [19], yet many teachers lack specialized training.

Insufficient involvement in various movement contexts is linked with several preventable non-communicable diseases, including cardiovascular diseases, diabetes, mental health problems, and obesity, that have a massive cost for both the individuals affected, their families, and society at large [20,21]. In turn, these illnesses disproportionally affect vulnerable groups and negatively affect long term marginalization outcomes such as educational attainment and work inclusion. Research to date has highlighted that life trajectories of marginalization and exclusion often have their onset in early childhood development, are comorbid across mental and somatic health, and are strongly heterogenic [22]. Social disparities in health manifest even before birth. For example, maternal health disparities can lead to pregnancy complications and poor birth outcomes such as a low birth weight, an important risk factor for developmental delays [23]. Such vulnerabilities may be compounded by academic and social problems, a difficult family situation, and environmental hazards [24,25,26] and result in long-term health issues, poor academic achievement, and unemployment [27,28,29].

This perspective paper argues for inclusion in motion, combining insights from developmental science, resilience research, public health, biological and genetic perspectives, and systems thinking to address long-term inequalities in health, development, and participation. By framing PA not just as a health behavior, but as a developmental right and a context for inclusion, we emphasize the role of movement-based settings in interrupting marginalization trajectories. These settings, such as PE, outdoor life, and organized or informal sports, can be leveraged not only to increase activity levels, but to foster agency, belonging, and competence among children and youth. Inclusion in motion thus requires a paradigm shift: moving away from focusing narrowly on increasing participation in PA to creating environments where diverse bodies and backgrounds are welcomed, supported, and empowered. This approach prioritizes long-term developmental gains over short-term performance outcomes and sees PA as a socially, biologically, and emotionally embedded experience. Through this lens, movement becomes both a means and a metaphor for inclusion—dynamic, relational, and essential to human development. This perspective paper aims to conceptualize how inclusive movement contexts can interrupt trajectories of marginalization and promote developmental equity, drawing on insights from developmental systems theory, resilience research, and interdisciplinary public health frameworks.

## 2. Conceptual Frameworks

**Figure 1:**
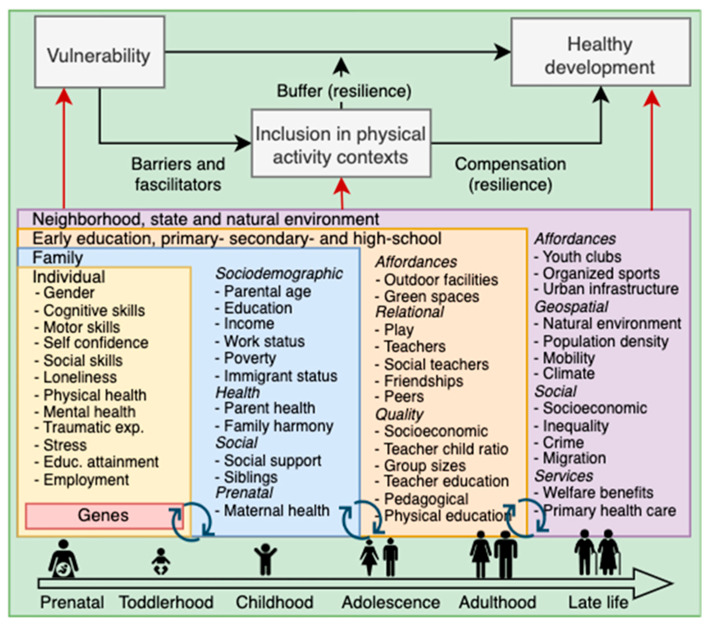
Inclusion in physical activity contexts as a buffer and compensatory factor across the life course. Black arrows represent potential causal effects, while red arrows represent selection effects and potential confounding effects.

The effects of time-limited preventive interventions in educational settings on children’s health and development tend to fade out over time [30,31]. The Center for Disease Control and Prevention and the WHO have, therefore, endorsed the importance of evidence-based prevention measures that target the wider set of forces and systems driving inequalities in health and well-being and shaping the conditions of daily life from an early age where people are born, grow, and live their daily lives [32,33]. As such, educational settings, organized sports, and nature-based recreation represent critical arenas for promoting inclusion and preventing marginalization. However, simply mandating increased levels of PA is unlikely to be sufficient. There is a growing need to move beyond traditional approaches that emphasize minimum activity thresholds or performance metrics. Instead, inclusion-focused strategies should adopt a more holistic model that integrates PA with psychosocial well-being. This implies designing interventions that foster long-term enjoyment and sustained engagement in movement-based activities, particularly for individuals from vulnerable or marginalized groups. To be effective, such interventions must be tailored to the diverse needs and lived experiences of participants and address barriers and facilitators at multiple levels: individual (e.g., low self-efficacy and mental or physical health challenges), social (e.g., a lack of peer support or exclusionary group dynamics), institutional (e.g., rigid curricula, undertrained teachers, or limited resources), and environmental (e.g., a lack of safe play spaces, geographic inaccessibility, or climate conditions). In doing so, inclusive movement contexts can have lasting impacts on participation, health, and belonging across the life course (Figure 1).

### 2.1. Developmental Systems Theory

Processes unfolding across time are the primary drivers of vulnerability, marginalization, and social disparities across life, and a relational developmental systems model is pivotal in exploring the immediate intricate interactions between children and their environments. As outlined by Lerner [34], this approach integrates the genetic, biological, psychological, and social facets of a person with environmental influences, viewing them as interconnected and mutually influential systems. These systems interact dynamically, creating a rich learning environment where people evolve together. Thus, it is important to recognize the diversity and plasticity of children and adolescents, suggesting that learning and development are not linear processes, but involve complex interactions within several upbringing settings. In accordance with most contemporary models of healthy development [35,36], the life-course model of development emphasizes that these processes continue throughout life, resulting from multilevel, adaptive, and bidirectional interactions between children and their physical and social environments [37,38]. Vulnerability, marginalization, and social disparities emerge from these interactions.

### 2.2. A Resilience Perspective on Vulnerable Children and Adolescents

The resilience framework is essential for understanding vulnerability and how to overcome the barriers that socio-economically and marginalized children and adolescents face [34,39,40,41]. Resilience refers to the biological, psychological, and social processes that enable individuals to adapt positively in the face of adversity [42]. Strengthening such protective factors, such as social support, self-efficacy, and access to enabling environments, can help compensate for and buffer against vulnerabilities [43]. This perspective is forward-looking, emphasizing children’s strengths, agency, and capacities rather than focusing solely on risk or deficits. Importantly, marginalization is rarely the result of a single event; rather, it often unfolds gradually through reciprocal dynamics, where risk in one domain undermines functioning in others [44,45]. Physical movement contexts, such as early childhood education, PE, formal organized sports, as well as informal negotiable movement activities and outdoor life, are key developmental arenas that can interrupt or exacerbate these trajectories. Barriers to participation in these contexts may reinforce existing vulnerabilities, creating downward spirals of exclusion. Conversely, inclusive experiences in such settings can initiate resilience cascades, where gains in physical competence, confidence, and social connectedness strengthen adaptive capacities. The accumulation of protective experiences across development may, therefore, play a crucial role in reducing long-term marginalization [39].

### 2.3. A Multidisciplinary and Multi Methodological Perspective

The complexity of processes at play discussed thus far requires a multidisciplinary and multi-methodological approach. This aligns with international frameworks, including the WHO’s Global Action Plan on Physical Activity [7] and the UN Sustainable Development Goals [46], which consistently emphasize the need for interdisciplinary and multisectoral approaches to promote inclusion, reduce inequalities, and support health and well-being across the life course. In line with these priorities, there is a growing recognition of the importance of integrating perspectives from developmental science, educational science, sports science, genetics, sociology, public health, and spatial ecology to better understand how life trajectories unfold. To address the complexity of inclusion and marginalization processes, a combination of qualitative and quantitative methods is essential. Qualitative insights, such as those derived from stakeholder interviews or focus groups, can help identify relevant mechanisms and experiences that may inform causal hypotheses. These can then be tested in larger-scale, population-based datasets. Conversely, quantitative findings can be followed up with in-depth qualitative exploration to better contextualize observed patterns. Such an iterative, mixed-methods approach increases both the scientific validity and the real-world relevance of interventions, ensuring they are grounded in both evidence and lived experience.

## 3. Strategic Approaches to Inclusive Physical Activity

### 3.1. Key Actors and Arenas for Action

Inclusion in motion, engaging more children in movement contexts, and preventing marginalization necessitates a multifaceted approach that includes enhancing accessibility to PA, creating inclusive and supportive environments for diverse groups, and integrating inclusive PA into education and broader health and social policies [37,47]. Early education teachers and schoolteachers are in a pivotal position to potentially reach all children and adolescents. Social education workers and clinical psychologists are key agents in helping vulnerable individuals and for promoting inclusion in schools. Sports coaches play a vital role in fostering inclusion and preventing dropout from organized sports, since the vast majority of children participate in, and drop out from, a sports club [48]. Outdoor educators are in a position to promote inclusion in outdoor life, an important arena for PA but with large social inequalities in participation [5]. Educational systems, community organizations, and public health actors are collectively positioned to promote inclusion in PA across key developmental arenas, such as early childhood education, schools, organized sports, and outdoor life.

### 3.2. Describe, Understand, and Intervene

**Figure 2:**
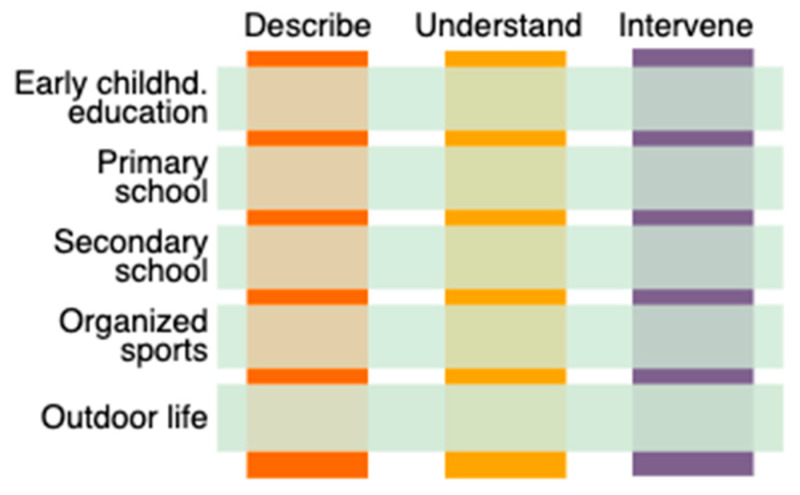
Upbringing arenas for inclusion in physical activity.

Efforts should especially focus on children and adolescents at heightened risk of marginalization due to factors such as a socioeconomic disadvantage, disability, refugee or immigrant background, minority status, gender identity, mental health challenges, or exposure to trauma [49]. The describe, understand, and intervene (see Figure 2) steps can guide such efforts. First, it is essential to systematically identify and describe existing inequalities, barriers, and facilitators for participation in PA across different settings and developmental stages. Second, there is a need for deeper understanding of the causal processes through which vulnerability and PA interact over time to influence long-term outcomes such as health, educational attainment, and work inclusion. Third, targeted interventions must be developed to address these barriers and disrupt potential cascades of disadvantage, ideally through context-sensitive and participatory approaches that involve stakeholders across sectors.

## 4. Foundations for Research and Action

### 4.1. What We Know: Foundations for Action

Inclusion in motion is a multidetermined, developmental process that unfolds from early childhood and across the life course. It is shaped by the interplay of genetic, biological, individual, family, and neighborhood factors [50,51]. Children with physically active parents are more likely to engage in movement-based contexts [52], and PA levels are moderately genetically heritable [53], indicating the relevance of both environmental and biological scaffolds.

At the same time, natural environments amplify the benefits of movement for health and development. Research shows that outdoor environments foster increased PA [54,55] and may enhance the psychosocial benefits of movement [56]. During early childhood, several studies have shown that motor skill development is closely linked to the affordances of outdoor environments. Parks [57] and woodlands with varied topography and vegetation [58] support exploration, risky play, and the development of gross motor skills, with measurable benefits for well-being [59].

However, movement contexts are not equally beneficial or accessible to all youth. Research shows that sport participants, particularly those involved in organized competitive youth sports, often report more positive attitudes and higher self-determined motivation in PE. These benefits tend to accrue to youth who are already confident, motivated, and supported in competitive contexts, suggesting that PE may unintentionally favor these groups and reinforce existing inequities [60,61,62]. Organized sports often exclude vulnerable youth, yet function as significant developmental assets for those who align with the demands and structure of the context. In contrast, inactive youth frequently face more complex psychosocial and environmental challenges than their more active peers [63,64], indicating that increasing PA per se should not be the sole aim of interventions. Instead, fostering emotional engagement and eagerness for movement may be a more developmentally appropriate target, especially for youth who are hard to reach through traditional PA arenas.

Informal, negotiable, and often self-organized lifestyle sport contexts, such as parkour, skateboarding, outdoor life, street basketball, and dance represent promising alternatives [65]. These arenas are often flexible and attuned to adolescents’ desires, identities, and social realities. Their humanistic and democratic character makes them particularly suitable for fostering inclusion, agency, and positive youth development. A recent review [60] found that nearly one-third of studies on lifestyle sports focused on youth from marginalized backgrounds, including those requiring physical, cognitive, or psychosocial support, disabled youth facing mental health challenges, and refugees or youth in contexts of conflict. This unexpectedly high number suggests that such movement contexts are increasingly viewed as pedagogically appropriate for supporting not only PA, but also everyday functioning and well-being. Taken together, these insights underscore that inclusive movement environments must be meaningfully tailored to the lived experiences, developmental trajectories, and support needs of diverse children and youth. Movement is not just a health behavior, it is a relational, developmental, and social experience that should be embedded in environments designed for inclusion, growth, and belonging.

### 4.2. What We Need to Discover: Knowledge Frontiers

Since PA participation is multi-determined, interdisciplinary ecological approaches that consider child, genetic, family, school, and environmental factors are called for [51]. Furthermore, we argue that there is a limited number of methodologically rigorous studies that disentangle genetic and environmental selection mechanisms, as well as spurious associations, from causal pathways [66]. Since a presumed causal risk and protective factors are often correlated, they should be investigated and adjusted for each other. For instance, PA during middle childhood is heritable [53], and shared traits may, therefore, operate as a 3rd variable in the association between parent and child PA levels. Moreover, few studies have explored action-oriented strategies for improving children and youth participation, social support, and health and wellbeing through sport and other movement activities. Qualitative approaches and strong partnerships are essential to understand the perspectives of key stakeholders. There is a shortage of intervention and experimental studies that integrate stakeholder knowledge to promote inclusion in these arenas. Researchers have, therefore, advocated for interest organizations and local governments to create collaboratives that include central actors such as schools and health and sport organizations [67]. Finally, there is a lack of intervention and experimental studies that examine the effects of PA interventions targeted towards vulnerable children and adolescents.

From a relational developmental systems perspective, future research should also investigate the developmental fit between individual needs and contextual affordances. While much attention has been given to the availability and structure of movement contexts, far less is known about how these contexts align with the motivations, psychosocial needs, and lived experiences of diverse youth. Vulnerable groups, such as those experiencing disability, trauma, exclusion, or marginalization, may encounter a mismatch between what is offered and what is developmentally supportive or meaningful. These misalignments can reinforce patterns of disengagement. To advance inclusive practice, research should move beyond supply-side logic and instead examine the dynamic and reciprocal relationship between what young people need and what they receive in terms of access, support, and perceived value in PA contexts. Studying this fit over time is crucial for designing interventions that are not only effective, but also equitable and developmentally grounded.

### 4.3. What We Need to Do: Methodological Approach and Research Execution

The endeavor to describe disparities and barriers to PA participation requires a multilevel and integrative approach, combining large-scale, longitudinal population studies with qualitative interviews and co-creation with key stakeholders. Comprehensive, genetically informed cohort studies that follow individuals from early childhood to adulthood are particularly valuable, as they can shed light on the potentially causal and dynamic mechanisms linking early vulnerability, inclusion in movement contexts, and long-term marginalization outcomes [68]. However, traditional statistical approaches offer a limited capacity to detect developmental cascades, that is, the recursive and accumulative processes through which risk and protection unfold over time [39]. To address this, novel analytical techniques are needed. These include random intercept cross-lagged panel models, which allow for bidirectional analyses of change while accounting for stable individual differences; longitudinal mediation models to examine indirect pathways of influence; and network analysis, which can model complex, mutually reinforcing relationships among vulnerability factors and movement participation patterns. Where relevant, polygenic risk scores can be used to adjust for genetic confounding and to explore gene–environment interplay.

Qualitative interviews are essential for capturing lived experiences, perceived barriers, and contextual facilitators that are not readily available in administrative or register data. Insights from these interviews can inform hypothesis generation, intervention design, and the interpretation of quantitative findings. Moreover, granular observational techniques, including video analysis or sensor-based data, can help assess the impact of pedagogical approaches on children’s engagement, movement quality, and sense of inclusion in situ. Physical fitness is increasingly recognized as a biomarker of health [69], and the correct identification of children’s fitness profiles may help educators, coaches, and health professionals individualize strategies that promote PA and resilience, particularly when such approaches are applied inclusively and with developmental sensitivity [70]. Finally, devising effective, context-sensitive interventions requires not only robust evidence, but also participatory, action-oriented designs. Collaborating with educators, coaches, health workers, and children themselves through co-creation and iterative testing ensures that interventions are both meaningful and feasible in real-world settings. In this way, an interdisciplinary and methodologically pluralistic framework can advance our understanding of how inclusion in movement contexts may help prevent marginalization and promote developmental resilience across the life course.

## 5. Research in Practice: An Interdisciplinary Model for Inclusive Movement Studies

### 5.1. The DECO Research Group: Integrating Systems Thinking and Inclusion

A growing body of interdisciplinary research is increasingly aligning with the developmental and systems-based approaches outlined in this perspective, combining insights from developmental psychology [34,39], life-course epidemiology [37], public health [32,33], and movement sciences [40,60,71]. The research group Development in Context (DECO) contributes to this field by examining how human development and lifelong learning unfold across diverse movement-related settings. These include formal education, PE, organized sports, informal and negotiable movement activities, outdoor recreation, rehabilitation, and self-directed movement activities. DECO brings together researchers with expertise in educational science, developmental psychology, motor development, outdoor studies, pedagogy, and public health. The group’s work is grounded in life course perspectives and relational developmental systems theory, emphasizing that development is a dynamic, context-dependent process shaped by the interplay between individual characteristics and environmental structures. To capture this complexity, DECO employs a wide range of methodologies, including quantitative longitudinal designs, geographic information systems (GIS), qualitative case studies, mixed-methods research, and advanced statistical modeling. This integrative approach enables the group to generate actionable knowledge about how inclusive movement environments can promote participation, belonging, and well-being across the lifespan. The group’s research collaborates with a broad set of stakeholders, including schools, municipalities, sports federations, outdoor organizations, and healthcare systems, with the aim of advancing equity in PA and reducing long-term marginalization.

### 5.2. Practice-Based Innovation: Akershus County as a Regional Driver

Akershus County, Norway’s most populous region, illustrates how regional governance can serve as both a driver and a partner in promoting equitable participation in PA among children and youth. With a dual mandate as both a formal administrative authority and societal developer, the county has taken an increasingly strategic role in addressing disparities in participation through long-term, system-oriented approaches. In recent years, Akershus has worked in close collaboration with researchers to ensure that its initiatives are evidence-informed and developmentally grounded. From 2025, Akershus County consolidated its inclusion efforts through three coordinated instruments. The first is a Youth Health Strategy, which emphasizes inclusive participation and mental well-being. The second is “Sport Without Barriers”, a flagship initiative aimed at removing structural and social obstacles in youth sport. The third is a strategic development program for sustainable and inclusive sports infrastructure, targeting underserved communities and marginalized groups. Together, these initiatives reflect a shift from fragmented programming to systems-level equity work, where PA is understood as both a developmental right and a tool for social inclusion.

Importantly, the approach taken by Akershus County is rooted in cross-sectoral collaboration and participatory governance. By engaging with youth, voluntary actors, municipalities, and professionals in education, sport, and health, the county enables the co-creation of environments where diverse young people can participate on different terms and in different communities. Through its collaboration with researchers, Akershus also contributes to a growing practice-based knowledge base that informs long-term learning and policy refinement. This partnership exemplifies how regional actors can align with the principles of relational developmental systems theory by creating structures that adapt to the evolving needs and lived experiences of children and adolescents. As such, the work undertaken in Akershus offers a replicable model for other regions aiming to embed inclusion in motion, integrating public policy, infrastructure development, and scientific insight to support thriving across the life course.

## 6. Discussion

While inclusive movement strategies hold great promise, realizing their potential in practice requires navigating persistent tensions. One such tension lies between universalistic public health messaging and the need for tailored, equity-focused approaches that recognize systemic barriers. Universal recommendations may fail to address the lived experiences of children and youth who face structural disadvantage, resulting in unintended exclusion [60,65]. Furthermore, there is a risk that current models of PE and organized sport reproduce dominant norms around ability, competition, and performance, which may alienate those who do not identify with such values. Another challenge concerns the gap between research and practice. Although developmental and public health frameworks emphasize contextual fit and adaptability, many interventions remain siloed within disciplinary or institutional boundaries. Translating knowledge into actionable, scalable solutions requires not only interdisciplinary research, but also strong implementation partnerships with educators, municipalities, and grassroots organizations. The Akershus County model illustrates how such alignment can be achieved, yet more evidence is needed to assess the sustainability and transferability of similar approaches across contexts.

This perspective underscores the urgent need to promote inclusion and reduce marginalization through movement-based contexts from early childhood through adolescence. PA is not only central to health promotion, but also a developmental resource that fosters resilience, agency, and belonging [40,42]. Yet, access remains unequally distributed, particularly for children and youth facing socioeconomic disadvantages, disability, or a minority status [7]. Addressing these disparities requires a systems-oriented, life course perspective that integrates insights from developmental science, education, public health, environmental studies, and policy. Inclusive strategies must move beyond universal recommendations and be tailored to diverse needs and lived realities. This involves co-developing solutions with stakeholders, leveraging natural environments, and embedding inclusive practices in schools, sports, and communities. The DECO research group illustrates how interdisciplinary and methodologically pluralistic research can support equity. In parallel, Akershus County shows how evidence can guide system-level strategies, combining health promotion, inclusive sport, and infrastructure development in a coordinated model. These partnerships offer a promising pathway for embedding inclusion in motion across sectors and over time. Future efforts should prioritize longitudinal, genetically informed research, stakeholder-driven interventions, and real-world testing, but also critical reflection on how institutional systems may reinforce or disrupt inequality through movement practices. Investing early in inclusive movement environments, and sustaining them through collaboration, offers a powerful opportunity to promote health, learning, and thriving across the lifespan.

### Future Directions and Concluding Remarks

The examples from DECO and Akershus County point toward concrete strategies that may inform both future practice and research. In practical terms, inclusive movement strategies should be embedded within educational and community infrastructures, co-developed with stakeholders, and adapted to local conditions and needs. Particular emphasis should be placed on reaching under-represented groups, strengthening inclusive pedagogical competencies among educators and coaches, and fostering low-threshold, socially supportive movement environments. From a research perspective, there is a need for longitudinal, genetically informed, and participatory designs that can clarify how PA contexts interact with developmental trajectories, especially for vulnerable youth. Further, studies should also examine how institutional and policy systems can evolve to ensure equitable access to meaningful movement participation across the life course.

## Data Availability

Data sharing is not applicable.
